# Supplementary material for: A Likelihood Approach for Real-Time Calibration of Stochastic Compartmental Epidemic Models
Source: PLoS Comput Biol. 2017 Jan 17;13(1):e1005257. doi: 10.1371/journal.pcbi.1005257 (PMC5240920; doi:10.1371/journal.pcbi.1005257)
Supplement: S1 File — (TAR.GZ) [file pcbi.1005257.s014.tar.gz › HSPH_Online-SI-Revision/MSS10/n1-extreme/n1-extreme_NEW_table0.pdf]

1	R0	{2.95019, 14.2854, 0.1}	{2.24323, 9.50287, 0.03}	{{2.12684, 8.23415, 0.}, {2.52588, 12.2207, 0.}}	{24.0155, 34.4179, 124.}	inf	302.	188.073	{150.649, 244.424}	37.7242	0.21821	newly	137.	129.84	{110., 150.}	9.64964	0.0445289	Reff	0.882107	0.917083	{0.85782, 0.970595}	5.01114	ar	924.	842.63	{764., 901.}	8.88636
2	R0	{2.67565, 16.946, 0.1}	{2.6337, 17.1139, 0.}	{{2.30907, 14.0824, 0.}, {2.93978, 19.9817, 0.}}	{10.2548, 14.7734, 100.}	inf	233.	208.922	{175.996, 234.933}	10.9258	0.0533243	newly	80.	77.85	{65., 89.}	9.3625	0.0419373	Reff	0.559211	0.60759	{0.565053, 0.644519}	8.77096	ar	926.	904.14	{864., 936.}	2.93305
3	R0	{2.40066, 16.6543, 0.1}	{2.29504, 14.5016, 0.22}	{{2.11132, 12.644, 0.}, {2.48143, 19.391, 1.}}	{9.5636, 18.0967, 290.}	inf	237.	214.104	{164.925, 273.199}	18.5245	0.0927163	newly	91.	101.86	{86., 116.}	14.5714	0.0580997	Reff	1.0178	1.01396	{0.948625, 1.08976}	4.3553	ar	916.	858.35	{809., 913.}	6.64956
4	R0	{1.9294, 15.4361, 0.1}	{2.26459, 16.9029, 0.99}	{{2.20881, 16.3002, 1.}, {2.46477, 18.9032, 1.}}	{18.0539, 12.5414, 900.}	inf	135.	188.336	{170.984, 208.93}	40.8479	0.147929	newly	58.	78.83	{68., 91.}	35.9828	0.130434	Reff	1.07853	1.14257	{1.08847, 1.22067}	6.46627	ar	796.	847.93	{812., 887.}	7.05905
5	R0	{2.92859, 19.5393, 0.1}	{1.67687, 7.30403, 1.14}	{{1.67405, 7.28757, 1.}, {1.68297, 7.28757, 2.}}	{42.7415, 62.6188, 1040.}	inf	261.	61.8561	{50.128, 71.8018}	76.3003	0.630137	newly	98.	51.07	{40., 60.}	47.8878	0.289039	Reff	0.66479	0.714527	{0.697612, 0.733019}	7.48168	ar	927.	674.32	{646., 709.}	27.2578
6	R0	{1.79622, 12.3148, 0.1}	{1.40864, 2.07742, 0.19}	{{1.39162, 1.89883, 0.}, {1.43965, 2.60601, 1.}}	{21.5776, 83.1307, 252.}	inf	97.	32.2018	{7.06835, 59.7648}	66.9151	0.603692	newly	44.	76.11	{13., 132.}	100.932	0.345007	Reff	0.941219	0.829283	{0.787355, 0.86142}	12.0644	ar	734.	498.84	{392., 592.}	32.0381
7	R0	{2.45855, 17.1776, 1.}	{1.92124, 11.4062, 2.09}	{{1.47488, 3.83841, 0.}, {2.12134, 14.6763, 3.}}	{21.8547, 34.4666, 171.}	inf	208.	134.957	{36.5707, 190.888}	35.152	0.271271	newly	81.	76.44	{52., 94.}	15.8272	0.0802028	Reff	0.98342	0.890536	{0.817375, 0.943877}	9.446	ar	871.	746.43	{539., 854.}	14.3295
8	R0	{2.20009, 15.519, 2.}	{2.01705, 13.609, 0.}	{{2.01705, 13.609, 0.}, {2.01705, 13.609, 0.}}	{8.31974, 12.3078, 100.}	inf	171.	127.623	{115.626, 138.676}	25.3664	0.128251	newly	72.	60.27	{49., 69.}	16.7083	0.082607	Reff	0.708429	0.736982	{0.713856, 0.760466}	4.13931	ar	839.	794.07	{758., 820.}	5.36234
9	R0	{2.06493, 9.6414, 1.}	{1.76848, 7.29809, 0.54}	{{1.44762, 3.83841, 0.}, {2.76849, 18.33, 2.}}	{27.5129, 62.4862, 100.}	inf	125.	109.394	{35.2516, 280.541}	67.4392	0.356439	newly	96.	91.27	{49., 121.}	24.1771	0.125586	Reff	1.08409	0.911666	{0.843544, 1.02049}	15.9102	ar	777.	651.81	{498., 913.}	24.3282
10	R0	{2.60775, 19.6936, 1.}	{2.24797, 18.4396, 0.04}	{{2.27582, 18.9086, 0.}, {2.27582, 18.9086, 0.}}	{13.7965, 6.36766, 96.}	inf	215.	228.197	{219.791, 251.14}	12.0391	0.0600852	newly	62.	81.59	{71., 95.}	33.2419	0.123208	Reff	0.904889	0.745727	{0.707144, 0.775186}	17.5892	ar	889.	872.41	{862., 900.}	2.46232
11	R0	{1.99168, 8.13555, 1.}	{2.63612, 17.1613, 1.61}	{{2.17882, 11.7575, 1.}, {2.86095, 19.9929, 2.}}	{36.7527, 120.011, 79.}	inf	102.	242.518	{178.613, 288.757}	148.038	0.406306	newly	81.	91.73	{78., 107.}	19.2963	0.0825327	Reff	0.938081	0.843475	{0.800022, 0.890872}	10.0851	ar	772.	888.31	{821., 944.}	19.2992
12	R0	{1.79304, 9.18941, 0.1}	{2.66569, 15.4449, 0.26}	{{2.14747, 10.7247, 0.}, {2.93488, 18.8445, 1.}}	{48.6688, 69.0312, 308.}	inf	134.	249.51	{172.958, 291.559}	86.5640	0.263879	newly	109.	115.6	{102., 129.}	9.59633	0.0398686	Reff	1.0059	1.17644	{1.09161, 1.2346}	16.9543	ar	725.	904.44	{836., 939.}	24.7503
13	R0	{1.84016, 18.3431, 1.}	{1.64851, 12.3239, 0.9}	{{1.38815, 6.71734, 0.}, {1.75663, 14.6725, 1.}}	{10.4151, 32.8147, 24.}	inf	100.	115.021	{39.5306, 157.852}	49.7511	0.235981	newly	41.	61.6	{38., 79.}	57.0732	0.197047	Reff	1.1354	0.982763	{0.929131, 1.02287}	13.3013	ar	762.	672.68	{480., 773.}	12.6457
14	R0	{1.94221, 9.85971, 1.}	{2.61601, 16.9934, 1.}	{{2.64334, 17.2462, 1.}, {2.64334, 17.2462, 1.}}	{34.6926, 72.3516, 0.}	inf	139.	220.836	{210.334, 235.057}	59.0646	0.199504	newly	92.	81.75	{69., 94.}	12.3587	0.0592072	Reff	0.594316	0.584175	{0.551402, 0.617657}	3.75268	ar	805.	910.19	{893., 929.}	13.0919
15	R0	{1.78578, 12.0541, 0.1}	{2.2194, 19.1478, 0.}	{{2.2194, 19.1478, 0.}, {2.2194, 19.1478, 0.}}	{24.2819, 58.8486, 100.}	inf	118.	171.168	{158.328, 183.874}	45.0572	0.160742	newly	65.	61.41	{51., 73.}	10.9385	0.0504245	Reff	0.878604	0.973945	{0.945742, 1.00239}	10.8515	ar	732.	835.29	{806., 863.}	14.1107
16	R0	{1.94463, 15.6503, 2.}	{1.91166, 10.2025, 1.29}	{{1.47488, 4.06661, 0.}, {2.1245, 13.1443, 2.}}	{10.9428, 35.7226, 35.5}	inf	143.	132.955	{40.7632, 176.372}	27.2642	0.167578	newly	51.	84.82	{63., 108.}	69.6078	0.227916	Reff	0.958703	0.947586	{0.877904, 1.00925}	4.56661	ar	757.	746.2	{549., 837.}	10.7107
17	R0	{2.08901, 16.4314, 0.1}	{1.70461, 9.56056, 0.09}	{{1.58886, 7.29751, 0.}, {1.71608, 9.75208, 0.}}	{18.4399, 41.9732, 172.}	inf	160.	96.4354	{79.1346, 113.609}	40.3646	0.229257	newly	61.	68.99	{54., 82.}	17.7541	0.0697768	Reff	1.04868	0.963222	{0.929941, 0.994345}	8.14934	ar	826.	680.74	{619., 738.}	17.707
18	R0	{2.76419, 18.56, 2.}	{2.65012, 16.7249, 1.05}	{{2.0968, 12.1899, 1.}, {2.93488, 18.8445, 1.}}	{9.9298, 11.3174, 47.5}	inf	275.	254.894	{166.298, 307.907}	16.0238	0.0846441	newly	103.	100.66	{81., 117.}	10.2524	0.0465427	Reff	0.931532	0.924882	{0.880409, 0.964235}	2.81355	ar	920.	899.87	{802., 946.}	4.49457
19	R0	{2.3821, 17.1399, 2.}	{1.95385, 11.393, 1.92}	{{1.44762, 4.00571, 0.}, {2.22867, 15.6411, 3.}}	{17.9935, 33.5294, 53.}	inf	229.	143.067	{45.3054, 204.835}	37.6592	0.267533	newly	77.	83.78	{58., 103.}	18.1299	0.0771208	Reff	1.07433	1.03039	{0.916096, 1.12333}	6.21848	ar	883.	747.89	{519., 866.}	15.3398
20	R0	{2.78314, 19.6127, 0.1}	{2.24242, 15.7579, 0.}	{{2.24242, 15.7579, 0.}, {2.24242, 15.7579, 0.}}	{19.4283, 19.6549, 100.}	inf	236.	120.743	{111.134, 129.682}	48.8376	0.291815	newly	84.	48.26	{41., 57.}	42.5476	0.24444	Reff	0.411905	0.590333	{0.57004, 0.611767}	43.3179	ar	943.	839.06	{816., 860.}	11.0223
21	R0	{1.85124, 14.6794, 0.1}	{2.02299, 17.0625, 1.45}	{{1.70573, 11.7348, 0.}, {2.20181, 18.765, 2.}}	{14.0026, 27.9134, 1372.}	inf	97.	130.937	{98.3835, 171.282}	44.0811	0.180148	newly	43.	55.59	{39., 73.}	35.4186	0.13079	Reff	1.20516	1.24102	{1.06571, 1.30355}	7.15581	ar	943.	772.06	{672., 848.}	12.6
22	R0	{2.1028, 14.9852, 2.}	{1.81696, 9.71526, 1.21}	{{1.77826, 9.06807, 1.}, {1.94282, 12.1446, 2.}}	{13.5932, 35.1676, 39.5}	inf	167.	102.853	{78.5127, 129.483}	38.4115	0.219404	newly	63.	65.56	{54., 77.}	11.4286	0.0474183	Reff	0.738083	0.752763	{0.724875, 0.773068}	2.76133	ar	840.	736.68	{683., 793.}	12.3
23	R0	{1.86601, 10.2157, 1.}	{2.225, 14.0257, 1.29}	{{1.77826, 9.06807, 1.}, {2.64334, 17.2462, 2.}}	{20.3668, 40.137, 29.}	inf	118.	190.525	{112.426, 261.176}	64.4332	0.20482	newly	79.	94.15	{75., 113.}	21.2532	0.080996	Reff	1.02071	1.04137	{0.985129, 1.09241}	3.74062	ar	747.	836.61	{748., 912.}	12.9839
24	R0	{2.34032, 10.0992, 0.1}	{2.47242, 11.8765, 0.02}	{{2.32891, 10.9956, 0.}, {2.52588, 12.5037, 0.}}	{6.64153, 18.7813, 116.}	inf	202.	218.238	{181.637, 242.185}	12.0476	0.0496965	newly	137.	120.37	{101., 137.}	12.9416	0.062362	Reff	0.840175	0.845735	{0.802027, 0.903491}	3.72536	ar	887.	883.28	{851., 911.}	2.01578
25	R0	{1.91548, 15.3401, 1.}	{1.97111, 13.5027, 0.41}	{{1.67732, 9.44073, 0.}, {2.19504, 17.1709, 1.}}	{7.64523, 14.6037, 59.}	inf	146.	141.51	{105.239, 171.218}	13.24	0.0617291	newly	68.	74.54	{62., 86.}	14.3824	0.058071	Reff	1.10523	1.14282	{1.04645, 1.21501}	5.44051	ar	765.	780.1	{690., 837.}	6.08105
26	R0	{2.75283, 10.6721, 0.1}	{2.07199, 7.77967, 0.18}	{{1.74592, 5.58477, 0.}, {2.19024, 8.31782, 1.}}	{24.7324, 27.3692, 244.}	inf	255.	152.29	{100.38, 183.715}	40.2784	0.236609	newly	153.	119.28	{100., 136.}	22.0915	0.113289	Reff	0.743264	0.768185	{0.726666, 0.803674}	4.60007	ar	926.	808.37	{729., 855.}	12.703
27	R0	{2.08367, 19.7849, 1.}	{1.55629, 9.69254, 1.22}	{{1.51295, 8.4085, 1.}, {1.66334, 14.5038, 3.}}	{25.31, 51.0104, 30.}	inf	158.	66.2283	{49.121, 99.1215}	58.0834	0.394295	newly	54.	43.74	{32., 54.}	20.037	0.103644	Reff	0.927233	0.834427	{0.815118, 0.852032}	10.0089	ar	833.	617.21	{557., 685.}	25.9052
28	R0	{2.64334, 17.1765, 0.1}	{2.63109, 17.809, 1.95}	{{2.21411, 14.2212, 2.}, {2.86095, 19.9929, 2.}}	{11.6599, 18.6175, 1850.}	inf	230.	240.367	{134.52, 290.898}	25.9238	0.120279	newly	99.	86.58	{68., 103.}	14.0606	0.0697169	Reff	0.734849	0.682527	{0.619499, 0.776156}	9.99299	ar	934.	899.44	{800., 950.}	4.72163
29	R0	{2.87008, 13.3872, 0.1}	{1.89096, 5.73313, 0.25}	{{1.737, 4.40955, 0.}, {1.9862, 6.28417, 1.}}	{34.1147, 57.1746, 308.}	inf	267.	103.385	{67.1129, 134.503}	61.2792	0.425995	newly	113.	103.23	{75., 127.}	14.7168	0.0716154	Reff	0.608457	0.708006	{0.673805, 0.744986}	16.3609	ar	941.	760.07	{693., 810.}	19.2274
30	R0	{2.06105, 19.6275, 1.}	{1.90731, 14.7042, 1.}	{{1.54207, 9.08517, 1.}, {1.97176, 15.6958, 1.}}	{7.45944, 25.0836, 0.}	inf	100.	54.6293	{27.3665, 67.4962}	45.3707	0.283741	newly	41.	24.08	{16., 32.}	41.2683	0.245212	Reff	0.715184	0.746478	{0.657214, 0.777352}	7.09496	ar	747.	701.48	{614., 747.}	6.31861
31	R0	{2.30764, 13.435, 2.}	{2.55303, 15.0741, 1.01}	{{2.17882, 11.7575, 1.}, {2.60786, 15.5928, 1.}}	{12.2769, 15.7349, 49.5}	inf	231.	229.486	{187.154, 257.243}	8.31656	0.0373057	newly	94.	99.45	{87., 111.}	9.64894	0.0401303	Reff	0.717676	0.793554	{0.749814, 0.832407}	10.6301	ar	877.	898.11	{863., 927.}	3.3854
32	R0	{2.30565, 11.9237, 0.1}	{2.50706, 14.7548, 1.}	{{1.74092, 5.58477, 0.}, {2.90165, 19.9929, 2.}}	{21.211, 45.8082, 942.}	inf	219.	225.073	{81.6544, 288.306}	32.8966	0.168475	newly	128.	99.25	{82., 115.}	22.5078	0.114801	Reff	0.788532	0.805057	{0.75378, 0.841048}	3.82502	ar	853.	865.67	{692., 943.}	10.6084
33	R0	{2.24031, 18.582, 1.}	{2.20046, 18.9203, 0.}	{{2.2194, 19.1478, 0.}, {2.2194, 19.1478, 0.}}	{1.77888, 3.78147, 100.}	inf	205.	212.771	{198.691, 228.119}	6.33176	0.0269433	newly	78.	79.19	{68., 90.}	9.39744	0.0406737	Reff	1.10895	1.0715	{1.03736, 1.10221}	3.43553	ar	864.	853.61	{824., 879.}	2.15162
34	R0	{2.33306, 15.9759, 0.1}	{2.05294, 12.4032, 1.29}	{{1.77826, 9.06807, 1.}, {2.35866, 16.9062, 2.}}	{12.9311, 26.0161, 1192.}	inf	204.	165.94	{94.7861, 228.209}	25.5785	0.138919	newly	96.	87.62	{70., 101.}	12.0417	0.0584801	Reff	0.874898	0.837182	{0.799234, 0.869078}	4.4448	ar	864.	807.45	{709., 891.}	7.59836
35	R0	{2.48357, 18.3573, 1.}	{2.54578, 17.7276, 2.02}	{{2.01498, 13.1443, 2.}, {2.73517, 19.6025, 2.}}	{12.3905, 13.2055, 106.}	inf	218.	256.571	{154.846, 301.993}	31.2009	0.133512	newly	79.	97.05	{80., 114.}	25.6582	0.0980619	Reff	1.0282	0.935316	{0.894071, 0.98157}	9.04321	ar	835.	890.5	{812., 936.}	9.67665
36	R0	{2.59548, 19.9046, 0.1}	{2.09867, 13.6275, 0.}	{{1.83592, 11.1369, 0.}, {2.30108, 15.555, 0.}}	{19.1434, 31.5357, 100.}	inf	240.	164.254	{109.093, 207.083}	31.5609	0.18058	newly	83.	79.12	{63., 93.}	11.7349	0.0545126	Reff	0.931777	0.902835	{0.865446, 0.938007}	3.59336	ar	863.	808.25	{707., 875.}	7.10892
37	R0	{2.29641, 16.9868, 0.1}	{1.86989, 15.0665, 0.}	{{1.8951, 15.9062, 0.}, {1.8951, 15.9062, 0.}}	{18.5732, 11.3044, 100.}	inf	186.	113.339	{102.599, 130.089}	39.0649	0.244383	newly	65.	49.73	{40., 60.}	24.2615	0.13118	Reff	0.803743	0.788143	{0.76726, 0.813537}	2.50391	ar	877.	753.26	{717., 793.}	14.1095
38	R0	{2.65743, 15.4569, 1.}	{2.22569, 14.7755, 0.32}	{{2.11292, 12.9719, 0.}, {2.46533, 18.6082, 1.}}	{16.2465, 17.4564, 68.}	inf	233.	178.356	{152.891, 215.285}	23.4523	0.119533	newly	114.	79.25	{67., 91.}	30.4825	0.160853	Reff	0.733451	0.732527	{0.704832, 0.762662}	2.60865	ar	897.	850.26	{817., 899.}	5.40914
39	R0	{2.67953, 14.5132, 2.}	{2.92144, 18.3649, 0.01}	{{2.88523, 18.2127, 0.}, {2.96978, 18.8857, 0.}}	{9.74245, 27.4137, 99.5}	inf	233.	282.577	{255.515, 313.774}	21.9413	0.085338	newly	117.	107.9	{92., 124.}	11.2821	0.0525826	Reff	1.15488	1.11241	{1.02719, 1.18208}	4.88186	ar	894.	931.45	{915., 949.}	4.38591
40	R0	{1.94594, 11.928, 1.}	{1.40442, 2.124, 0.14}	{{1.39162, 1.89883, 0.}, {1.43965, 2.60601, 1.}}	{28.0306, 82.9827, 90.}	inf	115.	26.4288	{5.91119, 44.5708}	77.9719	0.759281	newly	66.	55.09	{12., 92.}	41.6212	0.276125	Reff	0.8387	0.728581	{0.700003, 0.752823}	13.1297	ar	796.	539.12	{471., 598.}	32.2889
41	R0	{1.66297, 10.0047, 0.1}	{1.46548, 5.42484, 0.46}	{{1.3618, 4.00571, 0.}, {1.47608, 6.00596, 1.}}	{14.4756, 53.7409, 480.}	inf	73.	41.7563	{16.6116, 53.0953}	59.0486	0.373992	newly	55.	47.25	{23., 73.}	31.	0.168367	Reff	0.964523	0.891291	{0.83683, 0.935496}	7.80995	ar	716.	517.98	{418., 593.}	29.3408
42	R0	{2.91568, 16.4453, 1.}	{2.69873, 16.6951, 1.85}	{{2.19696, 12.0512, 1.}, {2.97157, 19.3547, 2.}}	{9.4058, 17.8657, 91.}	inf	275.	255.573	{185.834, 305.82}	15.0414	0.0734886	newly	105.	103.58	{93., 114.}	6.93333	0.0305109	Reff	0.906776	0.875914	{0.821262, 0.924318}	4.70081	ar	919.	907.18	{845., 950.}	3.64091
43	R0	{2.55898, 15.8889, 0.1}	{2.43174, 13.7373, 0.14}	{{1.47608, 4.06661, 0.}, {2.6365, 15.4074, 1.}}	{8.66097, 15.2499, 212.}	inf	219.	220.937	{53.8842, 271.845}	22.7925	0.139642	newly	75.	107.15	{84., 124.}	46.4133	0.166099	Reff	1.07989	0.995446	{0.886112, 1.05332}	7.82656	ar	904.	855.6	{584., 928.}	6.86726
44	R0	{2.28649, 15.9017, 0.1}	{2.37435, 15.2028, 0.31}	{{2.29055, 13.7413, 0.}, {2.49092, 17.9391, 1.}}	{6.22555, 10.2892, 348.}	inf	219.	205.537	{185.497, 227.257}	8.15623	0.0402201	newly	76.	89.53	{78., 101.}	18.7763	0.0728117	Reff	0.756828	0.814434	{0.777215, 0.847094}	7.722	ar	880.	871.7	{843., 898.}	2.39318
45	R0	{2.65121, 18.6339, 0.1}	{2.55789, 14.4578, 0.67}	{{1.93419, 8.56054, 0.}, {2.98558, 18.1089, 1.}}	{13.7787, 22.557, 640.}	inf	260.	235.799	{145.235, 315.171}	23.9039	0.120887	newly	105.	111.63	{94., 129.}	11.0762	0.045702	Reff	1.01011	1.0097	{0.948788, 1.09472}	4.24419	ar	928.	878.08	{771., 944.}	6.37069
46	R0	{2.52291, 18.0251, 2.}	{2.67602, 17.6562, 0.}	{{2.11292, 12.9719, 0.}, {2.93978, 19.9817, 0.}}	{12.6142, 13.5528, 100.}	inf	228.	266.238	{185.407, 312.407}	27.7033	0.129899	newly	79.	99.21	{85., 113.}	28.4684	0.10993	Reff	0.915816	0.847362	{0.813093, 0.879706}	7.48929	ar	894.	902.35	{832., 949.}	5.53132
47	R0	{2.22618, 17.1225, 0.1}	{1.80922, 10.0436, 0.39}	{{1.44485, 6.00596, 0.}, {2.11292, 12.9719, 2.}}	{18.7289, 41.3425, 434.}	inf	176.	114.966	{69.1341, 173.512}	35.6168	0.221273	newly	67.	77.9	{61., 96.}	22.5373	0.0879733	Reff	1.15316	1.03874	{0.93873, 1.12437}	9.92225	ar	850.	712.73	{584., 830.}	16.1729
48	R0	{2.04405, 16.073, 1.}	{2.32729, 19.0151, 2.97}	{{2.12588, 19.2116, 3.}, {2.39393, 19.7817, 3.}}	{16.8514, 23.9385, 203.}	inf	137.	222.93	{209.166, 244.366}	67.0602	0.228337	newly	68.	79.21	{68., 92.}	18.3971	0.072047	Reff	1.11196	1.05883	{0.983602, 1.10324}	4.87731	ar	779.	867.74	{845., 901.}	13.4326
49	R0	{2.84396, 18.097, 1.}	{2.56598, 15.9747, 2.65}	{{2.43143, 15.4278, 2.}, {2.8833, 17.1847, 3.}}	{10.7262, 11.7273, 171.}	inf	289.	214.256	{184.135, 276.667}	26.0667	0.140147	newly	115.	88.25	{76., 103.}	23.487	0.119884	Reff	0.665487	0.78313	{0.719199, 0.830256}	17.7573	ar	952.	886.71	{852., 932.}	6.85819
50	R0	{1.85341, 18.0975, 4.}	{1.45267, 4.09473, 0.07}	{{1.43965, 3.83841, 0.}, {1.47608, 4.06661, 0.}}	{21.7744, 77.3741, 98.75}	inf	148.	30.8993	{13.3899, 46.0648}	79.1221	0.73303	newly	54.	45.86	{20., 69.}	30.5926	0.17763	Reff	0.850715	0.836348	{0.807445, 0.868691}	3.14478	ar	754.	513.77	{439., 586.}	31.8607
